# HMC: Hybrid model compression method based on layer sensitivity grouping

**DOI:** 10.1371/journal.pone.0292517

**Published:** 2023-10-09

**Authors:** Guoliang Yang, Shuaiying Yu, Hao Yang, Ziling Nie, Jixiang Wang

**Affiliations:** School of Electrical Engineering, Jiangxi University of Science and Technology, Ganzhou, Jiangxi, China; Michigan State University, UNITED STATES

## Abstract

Previous studies have shown that deep models are often over-parameterized, and this parameter redundancy makes deep compression possible. The redundancy of model weight is often manifested as low rank and sparsity. Ignoring any part of the two or the different distributions of these two characteristics in the model will lead to low accuracy and a low compression rate of deep compression. To make full use of the difference between low-rank and sparsity, a unified framework combining low-rank tensor decomposition and structured pruning is proposed: a hybrid model compression method based on sensitivity grouping (HMC). This framework unifies the existing additive hybrid compression method (AHC) and the non-additive hybrid compression method (NaHC) proposed by us into one model. The latter group the network according to the sensitivity difference of the convolutional layer to different compression methods, which can better integrate the low rank and sparsity of the model compared with the former. Experiments show that our approach achieves a better trade-off between test accuracy and compression ratio when compressing the ResNet family of models than other recent compression methods using a single strategy or additive hybrid compression.

## Introduction

With the development of deep learning, breakthroughs have also been made in the application of multi-task parallel execution to realize functions on edge devices, Examples include applications in computer vision and engineering [1–6]. These apps use multiple deep neural networks internally to collaborate on tasks ranging from speech recognition [7] to object detection [8, 9] to achieve state-of-the-art functionality. However, with the increasing scale of the deep learning model [10–12], its deployment on edge devices lacking in computing power becomes more and more difficult. The tens or even hundreds of millions of parameters bring great challenges to data storage. The development of multi-application and multi-model is greatly limited by the read delay of off-chip memory devices and edge devices’ lack of computing power [13]. This paper aims to reduce the number of model parameters and improve the speed of model computation.

Model compression is the main means to reduce the deep model size [14, 15]. Deep models are often over-parameterized, and redundancy often leads to huge storage and computing resource requirements. But this redundancy also provides an opportunity to compress the deep model. With the increasing demand for model reduction, model compression technology has developed rapidly. Network pruning [16–24], low-rank tensor decomposition [25–30], quantization [31, 32], distillation [33, 34], and popular compression methods [35, 36].

Low-rank tensor decomposition forces the low-rank decomposition structure on the weight of the neural network, which can compress the model parameters exponentially, but will lead to a significant loss of accuracy. The emergence of lightweight models such as MobileNet integrates low-rank components into model architecture design. Further compression of these models using other compression strategies can still achieve better results than the original model, such as network pruning. So, can the combination of low-rank decomposition and network pruning achieve the same or better results than using a single strategy on a general network architecture?

**Fig 1:**
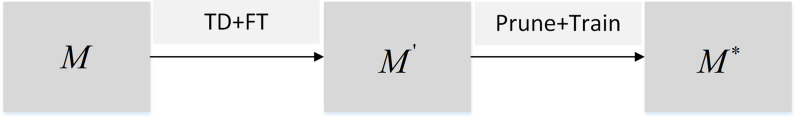
Additive hybrid compression method. Where TD stands for tensor decomposition and FT for fine-tuning.

In the existing work, some scholars have studied the compression of the VGG network [37] and ResNet network [38] by combining low-rank decomposition and sparse representation, while others have studied the low-rank + sparse weight compression of SOTA architecture that relies on efficient depth-separable convolution [39]. These methods apply additive low-rank plus sparse compression to the weights of the neural network, as shown in Fig 1, and can obtain better compression results than sparse compression or low-rank decomposition alone. The sparse compression here generally refers to the unstructured pruning strategy that uses a mask to carry out weight reset zero and freezing at the fine-grained level. The disadvantage of this method is that it has specific requirements on hardware and requires specific coding methods to achieve the purpose of reducing the demand for parameter storage.

**Fig 2:**
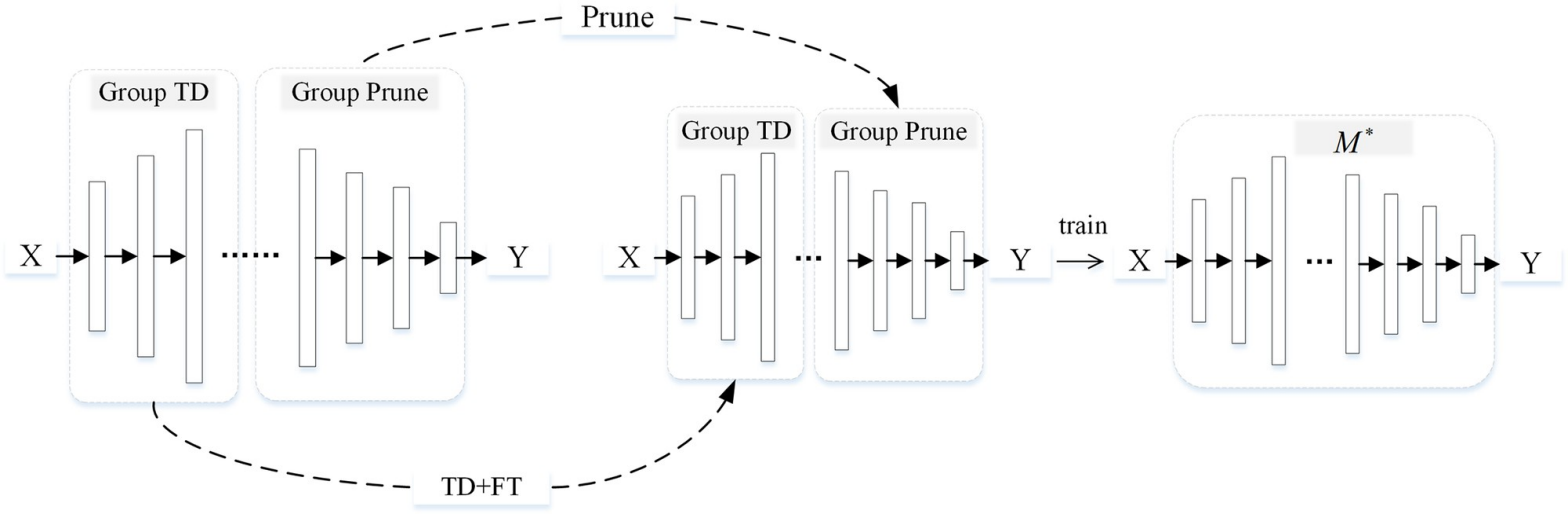
Non-additive hybrid compression method. Where TD stands for tensor decomposition and FT for fine-tuning.

In this work, we investigate the differences in sensitivity of low-rank decomposition and structured pruning to different convolutional layers in the baseline network and analyze the effects of different combination strategies on accuracy. Finally, a new hybrid model compression framework, called the hybrid model compression method based on sensitivity grouping (HMC), is proposed. Instead of sparse pruning, we use a more general and easy-to-implement structured pruning method and specifically design a general hybrid framework for low-rank decomposition and network pruning. In this framework, additive hybrid compression is a special case where the set of decomposition layers and the set of pruning layers are identical. The mixing strategy of the two compression methods can be changed by adjusting the set of network layers contained in the two groups. When layers using low-rank compression are completely different from those using pruning compression, it is called non-additive hybrid compression, which is shown in detail in Fig 2. In this case, the compression model tends to have the most competitive performance.

We summarize the contributions of this paper as follows:

1. To the best of the authors’ knowledge, our work is the first to investigate a model compression method that nonadditively mixes low-rank decomposition with structured pruning.
2. We find and use the sensitivity difference of convolutional layers to compression methods to group the models to select the most suitable hybrid compression strategy for the network.
3. We conduct extensive experiments on common architectures and benchmarks to show the superiority of HMC over other single or additive hybrid compression methods.

## Methodology

In previous studies, we often believe that even different compression methods (such as low-rank decomposition and pruning) can achieve almost the same results when compressing deep networks, so the weight to be compressed is usually approximated as the sum of low-rank and sparse components. However, in our experiments, we found a different conclusion. After using low-rank decomposition and structured pruning techniques to compress different layers of ResNet56, it is found that the same convolution layer produces different responses to different compression methods. Specifically, using low-rank decomposition to compress the first few layers of the convolutional neural network will hardly cause a loss of accuracy, but if the last few layers are compressed, the accuracy will decrease rapidly. Interestingly, pruning showed the exact opposite effect. Therefore, we design a hybrid model compression method based on layer sensitivity grouping.

### Grouping based on layer sensitivity

Convolutional neural networks perform multiple stages of reasoning on the input image, and each stage extracts features of different levels in the image, so different layers of CNNS often have different widths. This kind of change from the input end to the output end of the network is wide to narrow, and this change of structure leads to the convolution kernel parameters of different layers will adapt to different compression methods. First, we consider an L-layer convolutional neural network with the input image *x*, and the output feature of the layer *i* is *F*_*i*_:

$$F_{i}=x\prod_{l=1}^{i}W_{l},$$ (1)

Where $W_{l}$ represents the convolution of layer l, layer standardization, and activation function. For these three operations, we will not distinguish them separately in the subsequent derivation. The expression for wl is as follows:

$$W_{l}=ACT_{l}\times Norm_{l}\times Conv_{l}$$ (2)

Where *Act*_*l*_, *Norm*_*l*_, *Conv*_*l*_ represents the activation function, regularization function, and convolution operation of the layer respectively.

#### Layer sensitivity analysis

To measure the influence of different compression methods on model accuracy, sensitivity is defined as *SE*, the sum of reconstruction errors of output features of each layer of the network before and after compression. A deep network of layer *L*, *SE* is defined as:

$$SE=\frac{1}{2}{\sum_{i=1}^{L}\left\|F_{i}-F_{i}^{\prime }\right\|}_{F}^{2},$$ (3)

**Fig 3:**
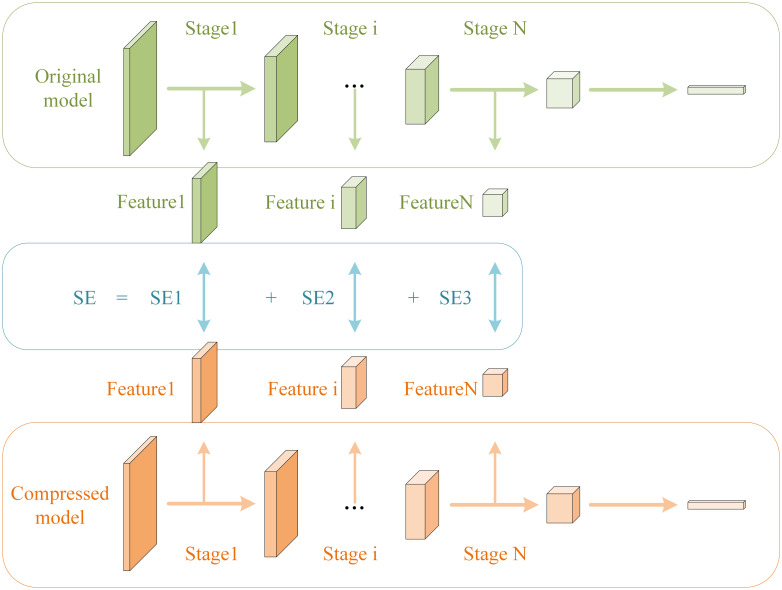
Illustration of the SE calculation process.

Where *F*_*i*_ represents the output feature map of the *i*-th layer of the original model, and $F_{i}^{\prime }$ represents the output feature map of the *i*-th layer after compression. As shown in Fig 3, we reshaped the middle feature map to a smaller size, which can effectively reduce the amount of computation for SE calculation.

The higher the sensitivity, the higher the precision loss caused by compression, we can measure whether a compression method is suitable for some layers of the network compression.

#### Network grouping

It is assumed that the pre-trained deep network has *L* convolutional layers, and $W_{i}$ is the parameter of the *i*-th convolutional layer. To select the most appropriate compression strategy for each layer, we divide all *L* layers of the network into two sets: $L_{l}=\left\{W_{l_{1}},W_{l_{2}},\cdots ,W_{l_{i}}\right\}$, where *l*_*i*_ ∈ {1, 2, 3, ⋯, *L*}; $L_{s}=\left\{W_{s_{1}},W_{s_{2}},\cdots ,W_{s_{i}}\right\}$, where *s*_*i*_ ∈ {1, 2, 3, ⋯, *L*}. All layers in *L*_*l*_ will be compressed using low-rank decomposition and all layers in *L*_*s*_ will be compressed using network pruning. When a layer exists in both *L*_*l*_ and *L*_*s*_, the layer is compressed using both methods. When all layers exist in *L*_*l*_ and *L*_*s*_ simultaneously, it is equivalent to the additive hybrid compression method. Reconstruct all weight tensors *W* as the sum of low-rank components and sparse components:

W=L+S, (4)

Where L represents the low-rank component and maintains the low-rank format; S represents a sparse component, which is obtained by structured pruning rather than sparse tensor format obtained by sparse mask.

In experiments, this format has the worst accuracy because most layers are not suitable for compression using both methods. In order to take full advantage of the different sensitivity of different network layers to different compression methods, we generally adopt low-rank decomposition for the front layer and network pruning for the back layer. Let *l*_*i*_ ∈ {1, 2, 3, ⋯, *L*}, *s*_*i*_ ∈ {*k* + 1, *k* + 2, ⋯, *L*}, where *k* is the boundary point between low-rank decomposition and network pruning, which is generally obtained by setting a sensitivity threshold.

**Fig 4:**
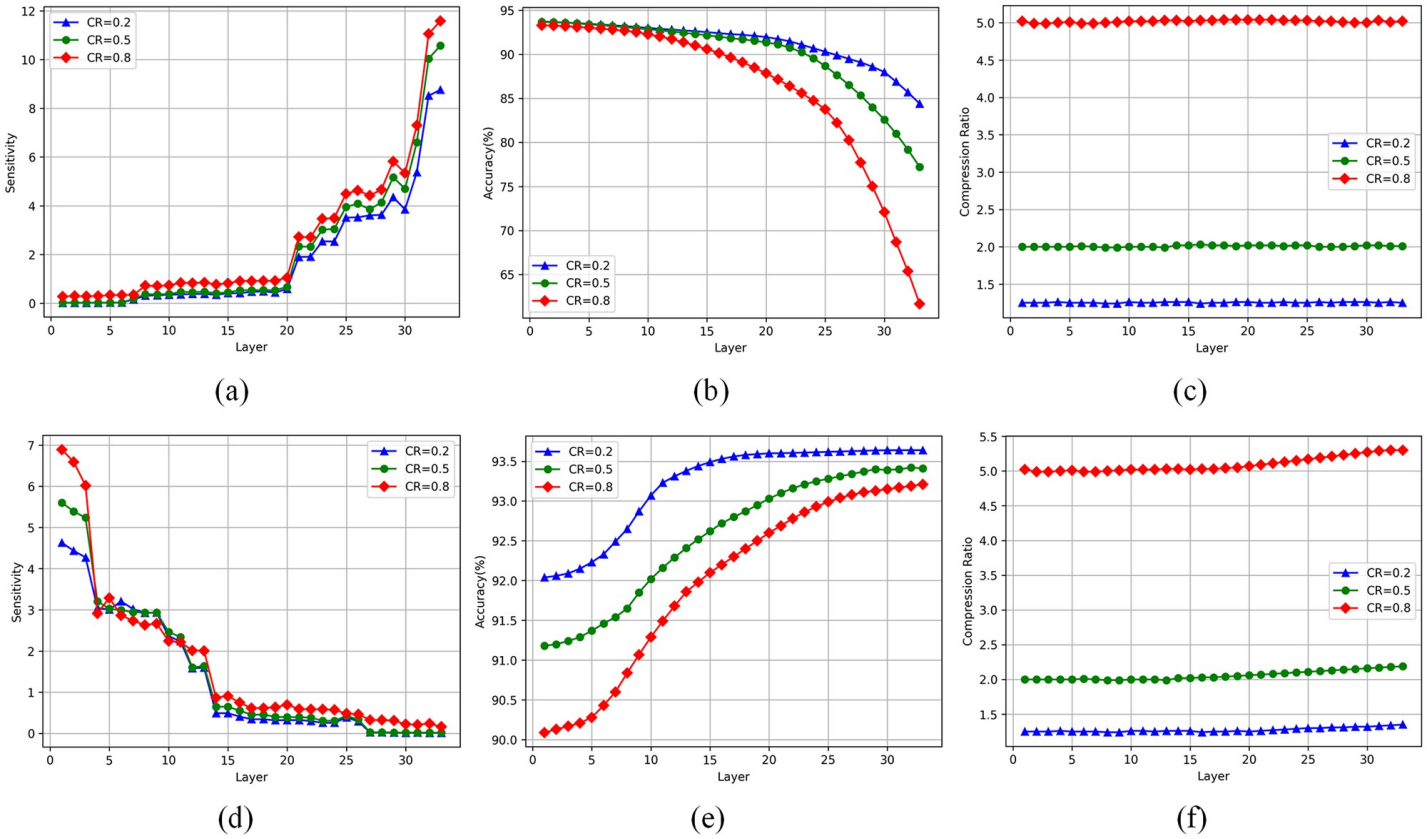
Effect of low-rank decomposition and network pruning on sensitivity and accuracy of different convolution layers under different compression ratios. (a) Sensitivity when using low-rank decomposition to compress different convolutional layers; (b) Accuracy when using low-rank decomposition to compress different convolutional layers; (c) Compression ratio of each layer in low-rank decomposition; (d) Sensitivity when pruning different convolutional layers; (e) Accuracy when pruning different convolutional layers; (f) compression ratio of each layer when pruning.

*k* is calculated by threshold method: Under the above conditions, the overlap degree of *L*_*l*_ and *L*_*s*_ decreases continuously, and the compression ratio of the model is unchanged, which makes the compression strategy transition from additive hybrid compression to non-additive hybrid compression. In this process, we find that the network reconstruction error *SE* shows a monotonically decreasing trend. At the same time, if we presuppose the boundary *k* value, it can be found that under the same compression ratio, *SE* presents an upward trend (with upper and lower limits) with the continuous increase of *k* value. Therefore, by setting the sensitivity threshold [*T*_min_, *T*_max_], the nearest *k* value can be taken as the boundary point between low-rank decomposition and network pruning when the model sensitivity meets *T*_min_ ≤ *SE* ≤ *T*_max_. According to Fig 4, the threshold value in the experiment is [5, 8].

### Non-additive hybrid model compression

The overall framework of the hybrid model compression algorithm can be expressed as:

$$\begin{array}{ll} M^{*} & =L\left(M_{L_{l}},\alpha \right)+P\left(M_{L_{s}},\beta \right)\\ s.t. & \left\{\begin{array}{c} \left|\alpha -1\right|\leq \delta \\ \left|\beta -1\right|\leq \delta \end{array}\right. \end{array},$$ (5)

Where, M* represents the compressed model; *L*(·) represents the low-rank tensor compression method, $M_{L_{l}}$ represents the part using low-rank decomposition in the deep model *M* to be compressed, and *α* represents the intensity of low-rank compression. *P*(·) Represents the structured network pruning method, $M_{L_{s}}$ represents the part of the deep model *M* that uses network pruning to be compressed, and *β* represents the strength of the network pruning method.

When *L*_*l*_ and *L*_*s*_ are completely coincident, the additive hybrid compression method can be expressed as:

$$\begin{array}{l} M^{*}=P\left(L\left(M,\alpha \right),\beta \right)\\ s.t.\;\left|\alpha +\beta -1\right|\leq \delta \end{array}$$ (6)

In this case, the models to be compressed are no longer grouped, which is equivalent to using two different compression methods to compress the models sequentially twice in additive hybrid compression. At this time, the compressed model loses high precision and competitiveness. We find that the compression effect is best when *L*_*l*_ and *L*_*s*_ are set as complementary states, that is, some convolutional layers in model *M* are compressed by low-rank decomposition, and the rest are compressed by network pruning. The compression mode at this time is called non-additive hybrid compression, and the boundary *k* value can be used to replace the expression of the set in the model. The model at this time can be expressed as:

$$\begin{array}{ll} M^{*} & =L\left(k⊙M,\alpha \right)+P\left(\left(1-k\right)⊙M,\beta \right)\\ s.t. & \left|\alpha +\beta -1\right|\leq \delta \end{array},$$ (7)

Where *k*⊙*M* represents the first *k* convolution layers contained in set *L*_*l*_ in the model *M*, and (1 − *k*)⊙*M* represents the remaining convolution layers contained in *L*_*s*_. At this point, the value of *k* can be obtained by setting the sensitivity threshold, and the grouping of each layer of the network can be completed. Besides, low-rank decomposition and pruning can be used to compress the layers in *L*_*l*_ and *L*_*s*_ respectively.

#### Algorithm

Our algorithm takes a pre-trained deep neural network *M* as input, and outputs a low-rank and sparse lightweight model M*, which is hybrid compressed by low-rank decomposition and network pruning. Firstly, the first step is to calculate the boundary *k* value according to the sensitivity threshold, and complete the model grouping. In the second step, low-rank decomposition is adopted for the layers belonging to the *k* set. The tensor rank set ${\left\{r_{i}\right\}}_{i=1}^{k}$ needs to be provided during decomposition. Finally, the layers belonging to the *L*_*s*_ set are pruned according to the pruning rate *s*, and the final output model M* is obtained. Table 1 summarizes the details of the non-additive hybrid compression algorithm.

**Table 1:** Algorithm 1: Nonadditive hybrid compression algorithm.

**Algorithm 1** Nonadditive hybrid compression algorithm
**Input**: pre-training network *M*; Sensitivity threshold [*T*_min_, *T*_max_]; Tensor rank ${\left\{r_{i}\right\}}_{i=1}^{k}$; Pruning rate *s*; Low order compression *e*_*l*_ epoch; After pruning train *e*_*s*_ epoch.
**Output**: Lightweight model M* satisfying compression rate
Calculate the boundary value *k* according to the sensitivity threshold [*T*_min_, *T*_max_], group the convolutional layers according to *k*, and obtain the sets *L*_*l*_ and *L*_*s*_. **for** *i* in *L*_*l*_ **do** The weight tensor *W*_*i*_ is low-rank compressed according to the tensor rank *r*_*i*_ of the *i*-th layer and *e*_*l*_ epochs of fine-tuning training for the network. **end for** **for** *j* in *L*_*s*_ **do** The weight tensor *W*_*j*_ was structurally pruned according to the pruning rate *s*. **end for** After pruning and *e*_*s*_ epochs training, the final lightweight model M* was obtained.

### Low-rank decomposition and network pruning

#### Low-rank decomposition

First, we standardize the description of the tensor format in this paper. This article uses a lowercase letter for a scalar, a bold lowercase letter for a vector, a capital letter for a matrix, and a bold uppercase art letter for a tensor. A tensor is a generalization of a matrix, or a multiplex array of data. An *n* order tensor is an *n*-dimensional array $A\in {\mathbb{R}}^{I_{1}\times I_{2}\times \cdots \times I_{d}}$, where *I*_*n*_ is the size of the *n*-th order and the (*i*_1_, *i*_2_, ⋯, *i*_*d*_)-th element of A is denoted as $a_{i_{1}i_{2}\cdots i_{d}}$. The number of terms in a tensor with a modal size of *n* and a modal number of *d* is *n*^*d*^. This exponential dependence on tensor order is often referred to as the "curse of dimension" and can lead to high costs in computation and storage. Fortunately, many practical tensors have low-rank structures and can take advantage of this property to reduce such costs. We will use Tucker decomposition and singular value decomposition (SVD) to reduce the parameters of the neural network.

Weight tensors in convolutional neural networks can be formally divided into two types: nuclear tensors with filter size 1×1 and nuclear tensors with filter size *k*×*k*(*k*>1).

*SVD decomposition*. Although the kernel tensor $A\in {\mathbb{R}}^{C_{in}\times C_{out}\times 1\times 1}$ with the size of filter 1×1 meets the format of a 4-order tensor in form, it can be operated in the form of second-order matrix $A\in {\mathbb{R}}^{C_{in}\times C_{out}}$ during decomposition. In addition, the weight of the FC layer can also be simplified to matrix operation. We use SVD decomposition to compress these weights.

#### Definition 1

SVD decomposition to decompose A 2-order matrix $A\in {\mathbb{R}}^{m\times n}$ into a diagonal matrix Σ and the product of a unitary matrix *U* and *V*:

$$A=U\Sigma V^{T},$$ (8)

Where $U\in {\mathbb{R}}^{m\times m}$, the eigenvector in *U* is also called the left singular vector of *A*; $\Sigma \in {\mathbb{R}}^{m\times n}$, the nonzero element on the diagonal of Σ is called the singular value of *A*; $V\in {\mathbb{R}}^{n\times n}$, the eigenvector in *V* is also called the right singular vector of *A*.

*Tucker decomposition*. For the weight tensor of filter size *k*×*k*(*k*>1), which satisfies the form of a 4-d tensor A, we use Tucker decomposition for compression. Tucker decomposition is a higher-order extended form of SVD. Tucker scheme extends the decomposition of 2-order matrices to *n*-order tensors. Next, we introduce a prerequisite definition for the lower-order Tucker format.

#### Definition 2

The product of mode *n* of tensor $A\in {\mathbb{R}}^{I_{1}\times \cdots \times I_{n}\times \cdots \times I_{d}}$ and matrix $U\in {\mathbb{R}}^{J\times I_{n}}$ is:

$$B=A\times_{n}U\Leftrightarrow b_{i_{1}i_{2}\cdots j\cdots i_{d}}=\sum_{i_{n}=1}^{I_{n}}a_{i_{1}i_{2}\cdots i_{d}}u_{ji_{n}}$$ (9)

The result is still a *d*-order tensor B, but the magnitude of the mode *n* becomes *J*. In the special case when *J* = 1, the *n*-th mode disappears and B becomes a tensor of order *n* − 1. The Tucker decomposition is defined by a series of pattern n-products between a small core tensor and several factor matrices:

#### Definition 3

Tucker decomposition represents the *d*-order tensor A as a series of pattern products of the core tensor G and factor matrix *U*.

$$A=G\times_{1}U^{\left(1\right)}\times_{2}U^{\left(2\right)}\times_{3}\cdots \times_{n}U^{\left(n\right)},$$ (10)

Where $G\in {\mathbb{R}}^{r_{1}\times r_{2}\times \cdots \times r_{d}}$ is the core tensor, the tuple (*r*_1_, *r*_2_, ⋯, *r*_*d*_) is the rank of Tucker’s decomposition, and $U^{\left(n\right)}\in {\mathbb{R}}^{I_{n}\times r_{n}}$ is the factor matrix of the *n*-th mode. When *r*_*n*_ = R, the compression rate of parameter number of Tucker decomposition reaches:

$$CR=\frac{R^{d}+R\sum_{i=1}^{n}I_{i}}{\prod_{i=1}^{n}I_{i}}$$ (11)

The product structure of Tucker’s decomposition allows for more expressive cross-dimensional interactions, but may not result in large parametric reductions due to the exponential dependence on the tensor *d*-order. Fortunately, we can achieve the desired compression ratio *CR* by adjusting the Tucker rank (*r*_1_, *r*_2_, ⋯, *r*_*d*_). Through Tucker decomposition, we decompose the 3*3 filters in the original network into a combination of {1*1, 3*3, 1*1}, which effectively reduces the number of parameters and storage requirements of the model.

#### Structured pruning

Structured pruning alters the structure of the neural network by physically removing model grouping parameters. Compared with unstructured pruning, structured pruning does not rely on specific AI accelerators or software to reduce memory consumption and computational costs. To highlight the superiority of the proposed method, a simple L1 norm criterion is used in this paper for pruning, and the same setting as DepGraph is used in the experiment [18].

The reason more robust pruning criteria and training techniques are not used is that the purpose of this paper is to provide a new compression method rather than an optimal strategy. However, our framework is generic for structured and even unstructured pruning which is common in the market [22–24], and using more advanced pruning criteria often results in better performance.

## Experiments

The experiment mainly includes two parts. First, we tested the sensitivity differences of convolutional layers at different locations to low-rank decomposition and structured pruning using the ResNet56 network officially provided by Torchvision. Secondly, to verify the validity of the proposed method, we apply our compression model to the image classification task on the ResNet series [40] and report its validation accuracy on the CIFAR/ImageNet dataset [41, 42]. All experiments in this section are based on the Pytorch implementation, the reference model in the experiments is from the official Torchvision implementation, and all experimental results are measured without using data enhancement.

Our experimental environment is as follows: The training equipment is Intel(R)Core(TM)i9-9900 processor, GeForce RTX2070SUPER(8G) graphics card; All experiments used pytorch1.11.0 framework and CUDA11.6+cudnn8.4.1 computing acceleration environment.

### Ablation study

In this section, we design three experiments to verify the influence of single and combined action of two compression strategies on network sensitivity, as well as the influence of different *k* values on network sensitivity. The experiments were conducted at three different compression ratios (*CR* = 0.2/0.5/0.8).

#### Sensitivity difference in a single compression method

Firstly, to study the sensitivity of different convolution layers in the network to a single compression method, 18 continuous convolution layers are obtained successively along the depth direction, and a single compression method is used for the experiment. In order to maintain a fixed compression rate, the rank of low-rank decomposition is appropriately expanded or the pruning rate is reduced in layers with a large number of channels. The experimental results of low-rank decomposition and network pruning are shown in Fig 4.

It is observed that for low-rank decomposition, the deeper layer is more sensitive, often accompanied by obvious accuracy loss, but the low-rank compression of the first few layers of the network can maintain good accuracy. Interestingly, the sensitivity of the convolutional layer to network pruning is exactly the opposite of that of low-rank decomposition: the overall sensitivity of the network decreases gradually as the compressed layer moves back, which indicates that the subsequent layer is less sensitive to network pruning, and the pruning technique will not lead to unacceptable precision reduction in general.

Do these results indicate that for ResNet56 networks, the convolutional layer at the front of the network is more suitable for compression using low-rank decomposition, while the layer at the back of the network is more suitable for compression using pruning techniques? A new question arises: Can the model maintain this characteristic when the two compression methods are applied simultaneously to the network?

#### SE when compressing with decomposition and pruning

**Fig 5:**
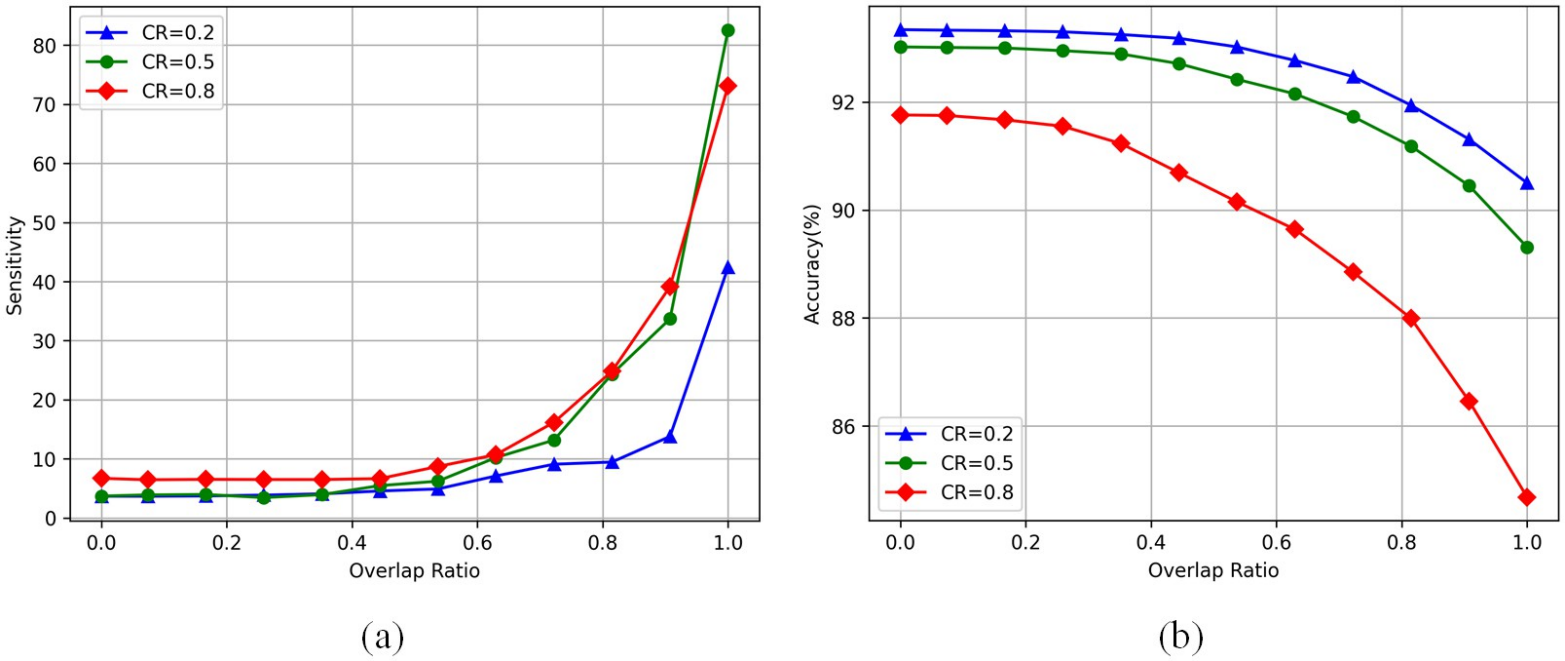
(a) sensitivity and (b) accuracy of the model when transitioning from the additive hybrid compression method to the non-additive hybrid compression method. The x coordinate represents the degree of overlap between the grouping sets *L*_*l*_ and *L*_*s*_.

We take the additive hybrid compression method as the starting point, and continuously reduce the overlap degree in sets until it is degraded to the non-additive hybrid compression method. It is worth noting that in this process, the low-rank decomposition tends to deal with the layer at the front of the network, while structured pruning is mainly used to deal with the layer at the back of the network. In this process, the rank set ${\left\{r_{i}\right\}}_{i=1}^{k}$ and pruning rate *s* of low-rank decomposition are constantly adjusted to keep the overall compression rate unchanged in each experiment. Fig 5(a) shows the changing trend of the overall reconstruction error of the network in the process of changing from an additive hybrid operation method to a non-additive hybrid compression method. It can be seen that as the compression method gradually degenerates to non-additive hybrid compression, the sensitivity of the network gradually decreases. This shows that our method selects a suitable compression strategy for each convolution layer, so that the compressed model retains a more effective structure and more information.

#### More experiments with reverse setting

**Fig 6:**
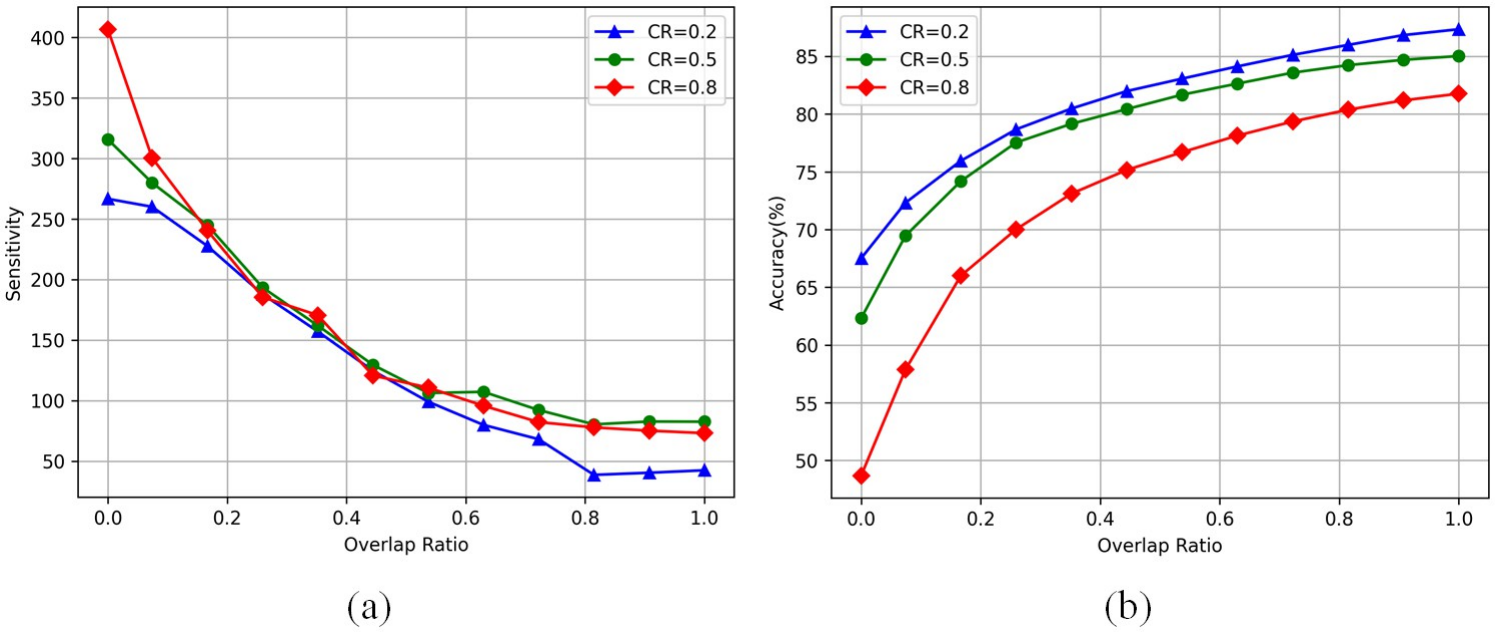
(a) sensitivity and (b) accuracy of the model when the additive hybrid compression method is transitioned to the non-additive hybrid compression method. In this case, the convolutional layer compressed by low-rank decomposition and network pruning is exactly the opposite to what we recommend.

We also set a contrast experiment which is opposite to this experiment. In the process of degradation, low-rank decomposition is biased to deal with the layer at the back end of the network, while structured pruning is used to deal with the layer at the front end of the network. Not surprisingly, with the gradual separation of the layers in the two grouped sets, the sensitivity of the network tends to rise rapidly, while the accuracy of the network decreases rapidly. The results of the variant experiment are shown in Fig 6.

#### Sensitivity difference at different *k* values

**Fig 7:**
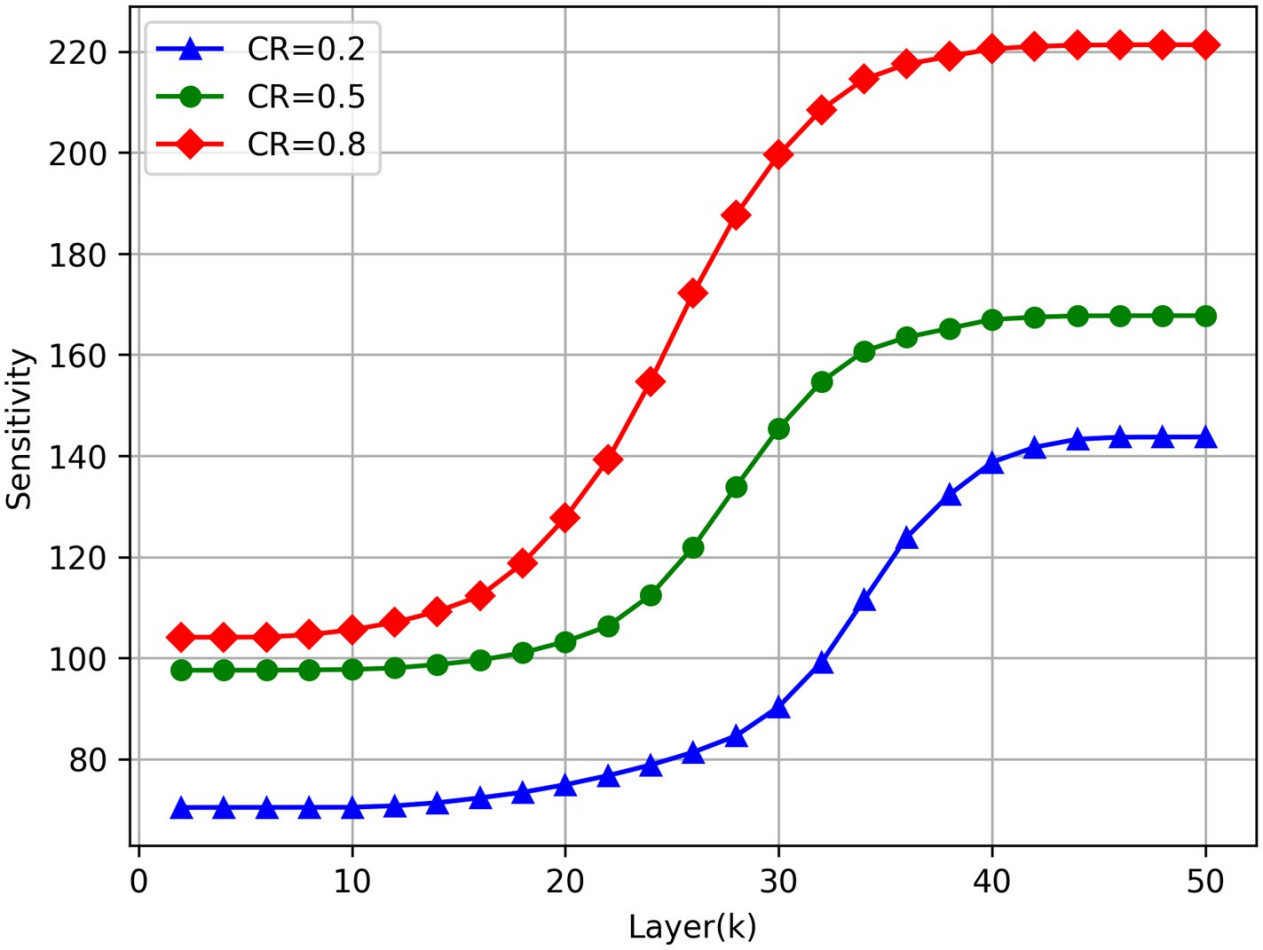
Sensitivity of the model when setting different k values and using HMC for compression.

At the same time, we further explore the influence of the boundary point *k* of the two compression methods on the sensitivity of the network. We pre-set different boundary point *k* values, which meet the characteristics that the boundary point moves gradually from the front end of the network to the back end. To make the experimental results more detailed, we assume that the boundary point was moved from the second layer to the 50th layer every four layers. For different preset boundary points, we also keep the compression rate of the model basically unchanged, but the experiment directly adopts the non-additive mixed compression method. The experimental results are also shown in Fig 6. It can be seen that the sensitivity of the network increases gradually as the boundary point moves back, and a stable state appears at the front and back layers. This indicates that the backward shift of *k* value will also lead to a monotonically increasing state of network reconstruction error, but there is an upper and lower limit in this process, that is, there is a range in the front and back layers of the network. within this range, the network sensitivity remains slowly changing or even unchanged, which provides an experimental basis for us to select the appropriate *k* value by sensitivity threshold method. At the same time, the existence of upper and lower limits also provides us with an effective candidate range of *k* value, and we can try our best to make *k* value fall in this range during the actual compression. As seen from Fig 7, the value of k has a low feature difference when it is [10, 18], and the boundary between pruning and decomposition is now clearer.

### Comparison of different compression methods

To verify the superiority of the proposed method, HMC is compared with the single compression algorithm and the additive hybrid compression algorithm, where tensor decomposition algorithms include Tucker [43], CP [44], TT [45], and MUSCO [28]. Structured pruning algorithms include Hrank [16], CHEX [17], DepGraph [18]; The additive hybrid compression algorithm includes literature [41] and ATMC [38]. These works represent the most advanced compression methods using a mixture of single structural assumptions and additive compression algorithms.

The experimental setup in this section is as follows: when training after pruning, the initial learning rate is 10e-2, and decreases to one-tenth of the original rate respectively at the 40/80 epoch. The training runs 100 epochs in total. Since our method belongs to the category of one-pass compression, the pruning rate s is set to be equal to CR. When using the low-rank decomposition compression network and making fine-tuning, we use a different learning rate: we set the initial learning rate to 10e-3 and drop tenfold at the third epoch. According to [45], We only have five epochs of fine-tuning. The tensor rank is set the same as [28].

#### Results on CIFAR10

**Fig 8:**
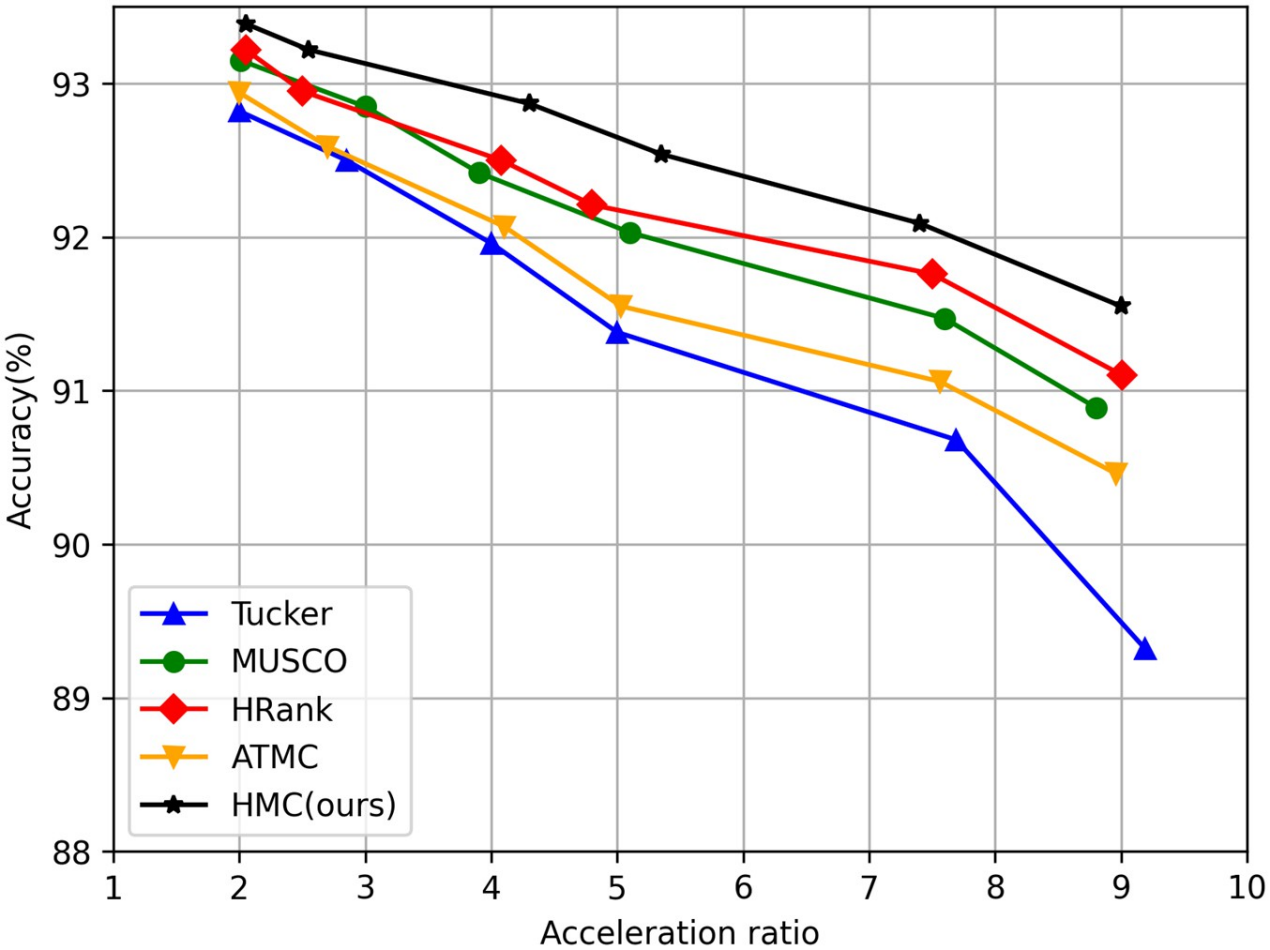
Trade-off curves of accuracy and acceleration ratio of different compression methods on ResNet56.

We show the trade-off curve of accuracy and compression ratio of ResNet56 based on various compression strategies in Fig 8, and report the experimental results in Table 2. We only compress the convolution layer because it contains most of the parameters of the network. We calculate the accuracy of the model when the compression ratio was 0.5/0.6/0.8, respectively. It can be seen that HMC can achieve higher accuracy with less storage cost at all compression ratios, while other compression methods will lead to significant performance degradation. For example, CHEX and DepGraph, the most advanced of pruning technologies, result in a 0.36% and 0.32% drop in ResNet56 accuracy at 2.53x and 2.57x compression ratios. However, our method only results in a 0.21% accuracy loss at a 2.55x compression ratio. Even when the compression ratio is as high as 5.35x, HMC only results in a precision loss of 0.89%, which is much lower than other methods at the same compression ratio (ATMC:1.88%; Literature [41]: 1.52%).

**Table 2:** Compression results of different methods on CIFAR-10. T-P: Hybrid model compression method combining low-rank decomposition and network pruning.

Model/Data	Method	Strategy	Speed up	Top-1(%)	Δ Acc
ResNet56	Baseline [26]	-	1.00 x	93.43	0.00
	Tucker [29]		2.00 x	92.82	-0.61
	TT [28]	TD	1.89 x	92.71	-0.72
	MUSCO [14]		2.01 x	93.15	-0.28
	HMC(ours)		**2.05 x**	**93.39**	**-0.04**
CIFAR10	Hrank [17]		2.50 x	92.95	-0.48
	CHEX [18]	Prune	2.53 x	93.07	-0.36
	DepGraph [19]		**2.57 x**	93.11	-0.32
	HMC(ours)		2.55 x	**93.22**	**-0.21**
	ATMC [24]		5.03 x	91.55	-1.88
	- [25]	T-P	5.11 x	91.91	-1.52
	HMC(ours)		**5.35 x**	**92.54**	**-0.89**

In order to verify whether more advanced network pruning strategies would benefit our approach, we used recently proposed structured pruning strategies [17–19] to replace the L1 criteria in the default methods, and examined the performance of compressed ResNet56 using these methods on CIFAR10. The results are shown in Table 3. Although L1 has achieved good performance (93.22%), HMC’s performance has been further improved by incorporating advanced pruning strategies. Especially after joining DepGraph [19], the model accuracy rate reached 94.02%.

**Table 3:** Results of different pruning strategies on CIFAR10. L1 is the default pruning standard used by HMC.

Method	L1	Hrank [17]	CHEX [18]	DepGraph [19]
Acc	93.22	93.28	93.71	94.02

#### Results on CIFAR100

In addition, we conduct experiments on a more complex benchmark dataset, CIFAR100, and report the results of compression experiments on ResNet32 based on various compression strategies in Table 4. Our method can also achieve relatively advanced accuracy at different compression ratios. For example, Tucker and CP decomposition brings 0.66% and 0.53% accuracy loss to the ResNet32 network at 2.01x and 1.99x compression ratios, respectively. The proposed HMC results in a precision loss of only 0.11% at 2.07x. When the compression ratio reaches 5.56x (higher than 5.36x and 5.50x in literature [38, 39]), HMC only results in 0.94% accuracy loss, which is much lower than ATMC’s 2.01% and 1.44% in literature [39]. This shows that our method achieves a higher compression ratio and better compression performance.

**Table 4:** Compression results of different methods on CIFAR-100.

Model/Data	Method	Strategy	Speed up	Top-1(%)	Δ Acc
ResNet32	Baseline [26]	-	1.00 x	68.82	0.00
	Tucker [29]		2.01 x	66.16	-0.66
	CP [30]	TD	1.99 x	68.29	-0.53
	MUSCO [14]		2.05 x	68.47	-0.35
	HMC(ours)		**2.07 x**	**68.71**	**-0.11**
CIFAR100	Hrank [17]		2.50 x	68.36	-0.46
	CHEX [18]	Prune	2.53 x	68.42	-0.40
	DepGraph [19]		**2.55 x**	68.51	-0.31
	HMC(ours)		**2.55 x**	**68.60**	**-0.22**
	ATMC [24]		5.36 x	66.81	-2.01
	- [25]	T-P	5.50 x	67.38	-1.44
	HMC(ours)		**5.56 x**	**67.88**	**-0.94**

#### Results on ImageNet

Table 5 shows the accuracy of compression of ResNet50 using different methods on Imagenet. When using low-rank decomposition alone or in combination with low-rank decomposition + pruning, our method forms a new SOTA. However, when the pruning strategy is used for compression alone, the accuracy of the model compressed by our method is lower than [18] (75.96%). We hypothesized that this was because we only used simple L1 criteria for pruning, and we did not use powerful training techniques in our experiments.

**Table 5:** Compression results of different methods on ImageNet.

Model/Data	Method	Strategy	Speed up	Top-1(%)	Δ Acc
ResNet50	Baseline [26]	-	1.00 x	76.32	0.00
	Tucker [29]		2.00 x	74.90	-1.42
	TT [28]	TD	1.89 x	74.47	-1.85
	MUSCO [14]		2.01 x	75.45	-0.87
	HMC(ours)		**2.05 x**	**75.71**	**-0.61**
ImageNet	Hrank [17]		2.50 x	75.60	-0.72
	CHEX [18]	Prune	2.53 x	75.91	-0.41
	DepGraph [19]		**2.57 x**	**75.96**	**-0.36**
	HMC(ours)		2.55 x	75.93	-0.39
	ATMC [24]		5.03 x	74.18	-2.14
	- [25]	T-P	5.11 x	74.57	-1.75
	HMC(ours)		**5.35 x**	**75.55**	**-0.77**

## Conclusions

We propose a hybrid model compression method (HMC) based on sensitivity grouping to take full advantage of the low rank and sparse characteristics of the model. We define the sum of reconstruction errors of the convolutional layer before and after compression as the model sensitivity, then group the models according to the sensitivity threshold method, and use different compression methods for different groups. The advantage of this is that the redundancy characteristics of the models are fully distinguished and utilized, and the accuracy loss caused by the additive mixed operation method is effectively avoided. Comprehensive experiments on the ResNet family of models demonstrate that our approach achieves a better trade-off between test accuracy and compression than other recent compression methods using a single strategy or additive hybrid compression. In future work, we will continue to study the unified framework of end-to-end deep compression. Specifically, we will further study the orthogonal compression strategies such as distillation and quantization, explore their independent redundancy characteristics, and fuse them into a unified compression model.
